# MARCH5-mediated quality control on acetylated Mfn1 facilitates mitochondrial homeostasis and cell survival

**DOI:** 10.1038/cddis.2014.142

**Published:** 2014-04-10

**Authors:** Y-Y Park, O T K Nguyen, H Kang, H Cho

**Affiliations:** 1 Department of Biochemistry, Ajou University School of Medicine, Suwon, Korea; 2 Department of Biomedical Sciences, Graduate School of Ajou University, Suwon, Korea; 3 Department of Physiology, Ajou University School of Medicine, Suwon, Korea

**Keywords:** MARCH5, Mfn1, acetylation, mitochondrial quality control

## Abstract

Mitochondrial dynamics and quality control have a central role in the maintenance of cellular integrity. Mitochondrial ubiquitin ligase membrane-associated RING-CH (MARCH5) regulates mitochondrial dynamics. Here, we show that mitochondrial adaptation to stress is driven by MARCH5-dependent quality control on acetylated Mfn1. Under mitochondrial stress conditions, levels of Mfn1 were elevated twofold and depletion of Mfn1 sensitized these cells to apoptotic death. Interestingly, overexpression of Mfn1 also promoted cell death in these cells, indicating that a fine tuning of Mfn1 levels is necessary for cell survival. MARCH5 binds Mfn1 and the MARCH5-dependent Mfn1 ubiquitylation was significantly elevated under mitochondrial stress conditions along with an increase in acetylated Mfn1. The acetylation-deficient K491R mutant of Mfn1 showed weak interaction with MARCH5 as well as reduced ubiquitylation. Neither was observed in the acetylation mimetic K491Q mutant. In addition, MARCH5-knockout mouse embryonic fibroblast and MARCH5^H43W^-expressing HeLa cells lacking ubiquitin ligase activity experienced rapid cell death upon mitochondrial stress. Taken together, a fine balance of Mfn1 levels is maintained by MARCH5-mediated quality control on acetylated Mfn1, which is crucial for cell survival under mitochondria stress conditions.

Mitochondria continuously change their network connectivity into fused or divided forms. Mitochondrial morphology *per se* is therefore the outcome of a balance between fusion and fission events. Mitochondrial fission is regulated by translocation of cytosolic Drp1 (dynamin-related protein 1) to mitochondria through association with the fission factors, Fis1 and/or Mff.^1, 2, 3^ Key factors in the fusion process include optic atrophy 1, the dynamin-related GTPase, located in the mitochondrial inner membrane as well as mitofusin1/2, localized to the outer membrane of mitochondria.^4, 5, 6^ Mfn1 and Mfn2 have 77% similarity at the amino-acid level and, however, they show tissue-specific differences in expression as well as in GTPase activities.^4, 5, 7^

The dynamic nature of mitochondria has a central role in preserving cellular homeostasis. Mitochondrial fusion allows damaged mitochondrial DNA (mutant mtDNA) to blend with intact mitochondria, thereby preserving mitochondrial function.^8^ Mutant mice lacking mitochondrial fusion activity show severe mitochondrial DNA mutations and depletions that precede respiratory defects.^9^ Fission events, on the other hand, generally facilitate apoptosis under high levels of cellular stress.^10^ Mitochondrial fragmentation promotes elimination of irreversibly damaged mitochondria through the process of mitophagy.^11^ Furthermore, cellular stress conditions such as oxidative stress, nutrient deprivation and others induce a transient change in the highly fused network morphology of the mitochondria. Mitochondrial hyperfusion has been postulated to be an adaptive response against diverse stress stimuli as mitochondrial hyperfusion sustains cell viability and improves energy supply.^12^ In part, mitochondrial hyperfusion induced by energy deprivation is mediated by phosphorylation on Drp1 and subsequent reduction of Drp1 levels.^13^ However, whether other cellular mechanism involving mitochondrial fusion molecules are related to this mitochondrial adaptation process has remained elusive.

The ubiquitylation–proteasome system related to the mitochondria regulates mitochondrial morphology and quality control.^14, 15^ In yeast, the Skp, Cullin, F-box-containing ubiquitin ligase, Mdm30p, has been shown to regulate mitochondrial fusion through degradation of Fzo1,^16^ and depletion of the deubiquitinating enzyme, USP30, induces mitochondrial elongation by increasing fusion activities in mammalian cells.^17^ A recent study also discovered two ubiquitylases, Ubp2 and Ubp12, that recognize ubiquitin chains on Fzo1 and act as quality control enzymes on the mitochondria.^18^ In mammals, mitochondrial ubiquitin ligase, membrane-associated RING-CH, MARCH5 (named MITOL), has been reported to regulate mitochondrial morphology through ubiquitylation of Fis1 and Mfn1 and 2, and mobilization of Drp1 from the cytosol to mitochondria.^19, 20, 21, 22^ Accordingly, depletion of MARCH5 triggers cellular senescence due to altered mitochondrial dynamics.^19^ Notably, MARCH5 also contributes to cellular homeostasis by targeting and degrading misfolded superoxide dismutase 1 and aggregated polyQ proteins that can cause mitochondrial damage,^23, 24^ accentuating its quality control function. The functional importance of ubiquitin ligase in mitochondrial quality control is highlighted by the cytosolic ubiquitin ligase, Parkin. Parkin is recruited to the mitochondria with low mitochondrial membrane potential and subsequently ubiquitinates Mfn1 and 2, triggering the elimination of impaired mitochondria.^25, 26^ A recent report identified the phosphorylated Mfn2 as a Parkin receptor on damaged mitochondria.^27^ Thus, the ubiquitylation–proteasome system in mitochondria contributes to mitochondrial dynamics and quality control, thereby having a central role in preserving cellular homeostasis.

In the present study, we discovered that MARCH5 serves as an upstream quality controller on Mfn1, preventing excessive accumulation of Mfn1 protein under stress conditions. We show that this MARCH5-dependent quality control on Mfn1 is crucial for mitochondrial homeostasis and cell viability.

## Results

### Mfn1 levels are elevated in cells exposed to AMA

**Figure 1:**
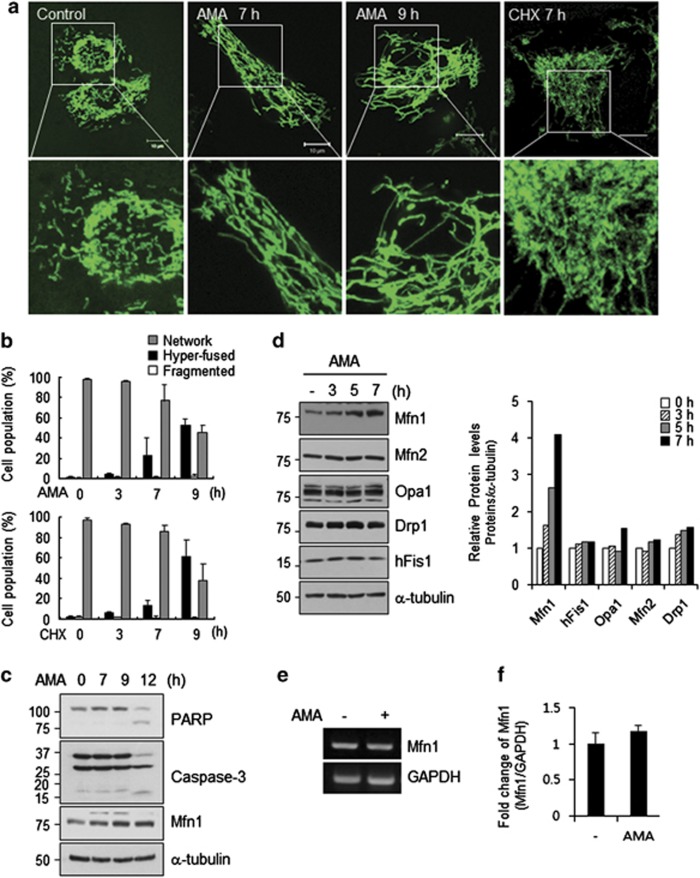
Mitochondria showed highly interconnected network structure in response to stress stimulus, AMA, accompanied by elevation of Mfn1 levels. After HeLa cells were treated with 10 *μ*M of AMA or 10 *μ*g/ml of CHX for indicated time, cells were analyzed. (**a**) After cells were exposed to stress stimuli (AMA, antimycin A; CHX, cycloheximide), HeLa cells were stained with MitoTracker Green and mitochondrial morphological change was observed on live cell imaging system. Images of representative fields were captured by confocal laser microscopy. Scale bars represent 10 *μ*m. (**b**) Quantification of mitochondrial morphology. Cell population represents percentage of cells population with normal (network), hyperfused (elongated) or fragmented mitochondria. At least 250 cells in several fields were counted in three independent experiments. Error bars, mean±S.E.M. (**c**) PARP cleavage or activation of caspase-3 was examined following AMA exposure in HeLa cells by immunoblotting for indicated time. (**d**) Total cell lysates were analyzed to determine the expression levels of mitochondrial dynamics controlling proteins by immunoblotting. (**e**) The mRNA levels of Mfn1 at 5 h after treatment of AMA were analyzed by RT-PCR. The templates were amplified for 30 cycles in each PCR reaction. (**f**) Expression of Mfn1 mRNA assessed by real-time PCR in untreated and AMA (5 h) treated HeLa cells. Fold change of mRNA of Mfn1 value was relative to untreated control and those were normalized by GAPDH. Data represent average of three independent experiments. Error bars, mean±S.E.M.

When cells are exposed to a variety of stresses, mitochondrial elongation or hyperfusion often occurs and is considered as an adaptive process.^12, 13^ However, the specifics of the involvement of mitochondrial fusion and fission molecules in this adaptation process are only partly understood. Here, we set up mitochondrial stress conditions using antimycin A (AMA), an inhibitor of electron transfer at complex III, and monitored the morphological changes of mitochondria in HeLa cells. As AMA is known to induce apoptosis,^28^ we first monitored the morphological changes of mitochondria under fluorescence time-lapse microscope after treatment with different concentrations of AMA (10–100 *μ*M).^29, 30^ Cells underwent rapid cell death when exposed to 50 or 100 *μ*M of AMA, whereas 10 or 20 *μ*M of AMA initiated mitochondrial hyperfusion and subsequent mitochondrial fragmentation (Supplementary Movies 1–5). Accordingly, mitochondrial hyperfusion was most prominent with AMA treatment in both classical HeLa (Figure 1a) and HeLa S3 cells (Supplementary Figure 1), a subclone of HeLa cells that shows more fragmented mitochondria in proliferating cells. Confocal microscopy images revealed that the mitochondrial hyperfusion reached a peak ∼9 h after AMA treatment; ∼60% of cells showed hyperfused mitochondria and fragmented mitochondria were also detected (Figures 1a and b). Similar to a recent report,^12^ cycloheximide (CHX) treatment resulted in hyperfused mitochondria that were even more tangled (Figure 1a; Supplementary Figure 2). The highly tangled mitochondrial network induced by CHX was observed in more than 60% of mitochondria at 9 h (Figure 1b). When we determined the cleaved poly (ADP-ribose) polymerase (PARP) as an apoptotic marker, an aberrant increase of the cleaved PARP was observed at 12 h after AMA (Figure 1c). A dramatic decrease of total caspase-3 levels was also observed at 12 h (Figure 1c), indicating that cell death occurred within 12 h of initiating AMA treatment. Thereafter, we continued the experiment using AMA.

We next examined whether the levels of mitochondrial dynamics proteins changed during the process of mitochondrial hyperfusion. Immunoblot analysis revealed Mfn1 levels go up approximately threefold (Figures 1c and d and Figure 3f). This increase in Mfn1 was first apparent at 5 h of AMA treatment (Figure 1d) and persisted up to 12 h (Figure 1c). In contrast, other mitochondrial morphology-controlling proteins were unchanged (Figure 1d). In addition, no change in Mfn1 mRNA level was detected (Figures 1e and f). Both data indicate that the increase in Mfn1 under mitochondrial stress is regulation at the post-transcriptional level. It was noteworthy that entangled mitochondrial hyperfusion induced by CHX treatment did not accompany Mfn1 accumulation suggesting that mitochondrial hyperfusion is induced by different mechanisms (Supplementary Figure 2). These data show that mitochondrial hyperfusion was accompanied by an elevation in Mfn1 levels in cells exposed to mitochondrial stress by AMA treatment.

### A balance in Mfn1 levels is crucial for cell survival under mitochondrial stress

**Figure 2:**
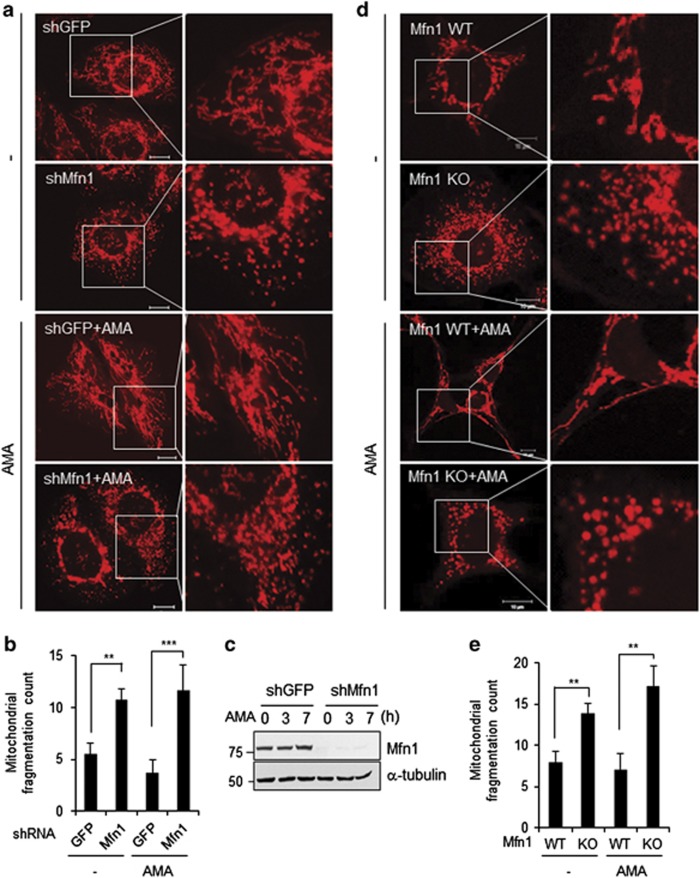
Mfn1 is required for mitochondrial hyperfusion under AMA-induced stress condition. HeLa cells were transfected with shGFP or shMfn1. The transfected cells were selected and further grown in presence of 30 *μ*g/ml of hygromycin B. (**a**) After GFP or Mfn1 shRNA expressing cells were exposed to AMA (10 *μ*M) for 5 h, mitochondria were stained with MitoTracker Red and cells were fixed. The images, of representative mitochondrial morphology, were analyzed by using confocal microscopy. Scale bars represent 10 *μ*m. (**b**) Acquired images (*n*=10) from **a** were background subtracted, filtered (median), thresholded and binarized to identify mitochondrial segments using ImagJ. The mitochondrial fragmentation count (MFC) was counted with the particle counting subroutine after normalization to total mitochondrial area (in pixels) as shown previously.^41^ Data represent average of three independent experiments. Error bars, mean±S.E.M. **P<0.01, ***P<0.001 *versus* shGFP by Student's *t*-test. (**c**) The expression levels of Mfn1 were determined by immunoblotting. (**d**) After Mfn1 WT or knockout (KO) MEF cells were exposed to 10 *μ*M of AMA for 5 h, mitochondrial morphology was analyzed by confocal microscopy. Scale bars represent 10 *μ*m. The mitochondrial images were captured in representative fields. (**e**) MFC was analyzed with acquired images (*n*=10) from **d**. Data represent average of three independent experiments. Error bars, mean±S.E.M. **P<0.01 *versus* MARCH5 KO by Student's *t*-test

**Figure 3:**
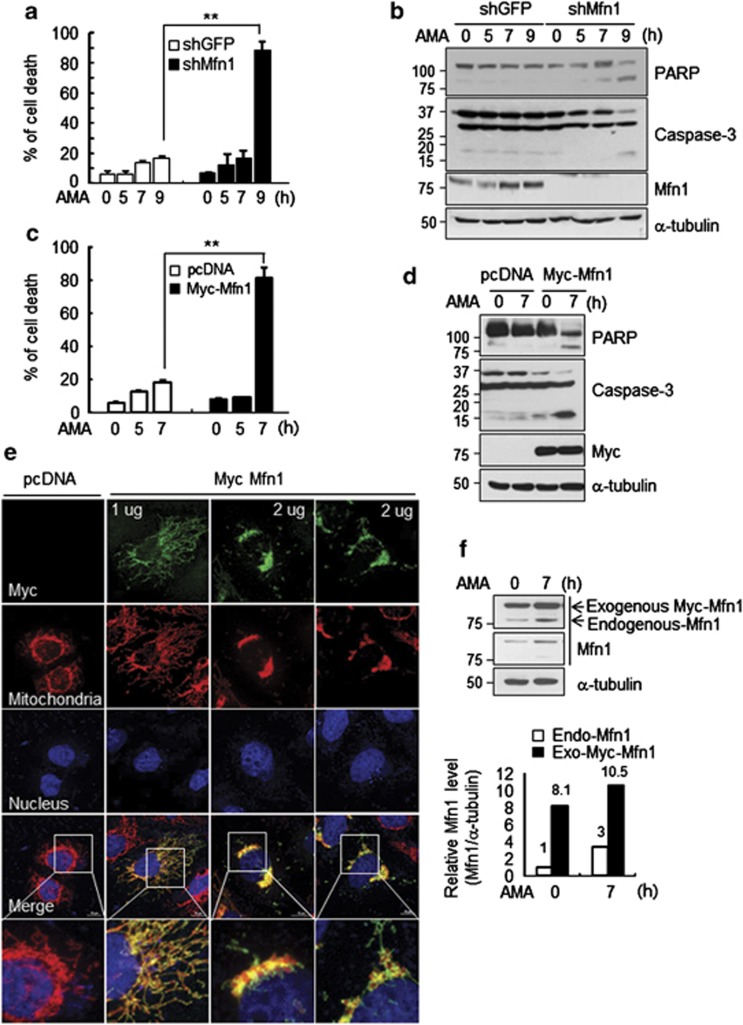
Maintaining a balance of Mfn1 levels is important to protect against stress-induced cell death. (**a**) After HeLa cells were transfected with shGFP or shMfn1, the transfected cells were selected and exposed to 10 *μ*M of AMA for indicated time, followed by cells being collected and stained with trypan blue. The calculated percentages of stained cells to total cells represent the percentage of dead cells and data represent average of three independent experiments. Error bars, mean±S.E.M. **P<0.01 *versus* shGFP by Student's *t*-test. (**b**) The lysates were obtained from **a** and PARP cleavage or activated capsase-3 levels were analyzed by immunoblotting. (**c**) After control vector (cDNA) or Myc-Mfn1 was introduced into HeLa cells, cells were treated with 10 *μ*M of AMA for indicated time and dead cells were counted. Data represent average of three independent experiments. Error bars, mean±S.E.M. **P<0.01 *versus* cDNA by Student's *t*-test. (**d**) PARP cleavage or caspase-3 activation was assessed by immunoblotting with the lysates obtained from **c**. **(e)** After HeLa cells (3 × 10^5^) were seeded on coverslip in 60 mm culture plate, two different doses (1 or 2 *μ*g) of Myc-Mfn1 were introduced. Myc-Mfn1-expressing cells were stained with MitoTracker Red (red) and DAPI (blue) for the mitochondria and nuclei, respectively. Myc-Mfn1 was detected by Alexa488 (green) -labeled secondary antibody. Representative mitochondrial images were captured by confocal microscope. Scale bars represent 10 *μ*m. (**f**) Comparison between the protein expression levels of Mfn1 (endogenous) and Myc-Mfn1 (exogenous) by using anti-Mfn1 antibody. The intensity of the bands was measured by Image-J software (NIH, Bethesda, MD, USA). Fold change relative to control was calculated. The graph represents quantification of Mfn1 expression levels by normalization to α-tubulin (bottom)

To determine the necessity of Mfn1 in mitochondrial hyperfusion, we depleted Mfn1 in cells. As shown in Figure 2c, Mfn1 shRNA efficiently reduced the levels of endogenous Mfn1 protein. Under a confocal microscopy, Mfn1-depleted cells displayed dot-like fragmented mitochondria (Figures 2a and b), as expected. Unlike control cells, Mfn1-depleted cells failed to undergo mitochondrial hyperfusion upon AMA treatment. Consistent with this finding, we failed to detect any mitochondrial hyperfusion in Mfn1^−/−^ mouse embryonic fibroblast (MEF) upon adding AMA (Figures 2d and e). Thus, the data indicate that Mfn1 is necessary for AMA-induced mitochondrial hyperfusion. We next addressed whether mitochondrial hyperfusion mediated through Mfn1 contributed to cell survival. In cells lacking Mfn1, 90% appeared to be dying 9 h after AMA treatment, whereas less than 20% of control cells were dying (Figure 3a). Consistent with this, the cleaved PARP first appeared at 5 h and became evident 7–9 h after AMA treatment in Mfn1-depleted cells, whereas no significant accumulation of cleaved PARP was found up to 9 h in control cells (Figure 3b). These data illustrate that Mfn1-mediated mitochondrial fusion activity is crucial for cell survival under mitochondrial stress.

We were curious that the increase in Mfn1 levels in response to AMA was consistent, but moderate. We therefore investigated whether cells expressing higher levels of Mfn1 protein, and thus had higher mitochondrial fusion activity, would show an improved survival response to AMA. Surprisingly, however, we observed that cells ectopically expressing Myc-Mfn1 experienced severe cell death, with ∼90% of cells dying within 7 h of AMA treatment (Figure 3c). These findings are consistent with the dramatic increase of cleaved PARP in Myc-Mfn1-overexpressing cells exposed to AMA (Figure 3d). Without AMA, overexpression of Mfn1/2 (2 *μ*g) showed a prominent perinuclear clustering of the mitochondria (Figure 3e; Supplementary Figure 3). The severity of mitochondrial clusters was correlated with the amount of Mfn1 introduced into cells (Figure 3e). We compared the expression levels of Mfn1, and found that the level of ectopically expressed Myc-Mfn1 in AMA-treated cells was much higher than that of endogenous Mfn1 regardless of AMA treatment (Figure 3f). Thus, it is likely that a moderate increase in Mfn1 under mitochondrial stress facilitates cell survival, whereas an excess accumulation of Mfn1 protein is detrimental to cells especially under mitochondrial stress. Therefore, it could be speculated that moderate Mfn1 levels under AMA treatment is achieved by upstream quality control systems.

### Mfn1 levels are controlled by MARCH5-mediated ubiquitylation

**Figure 4:**
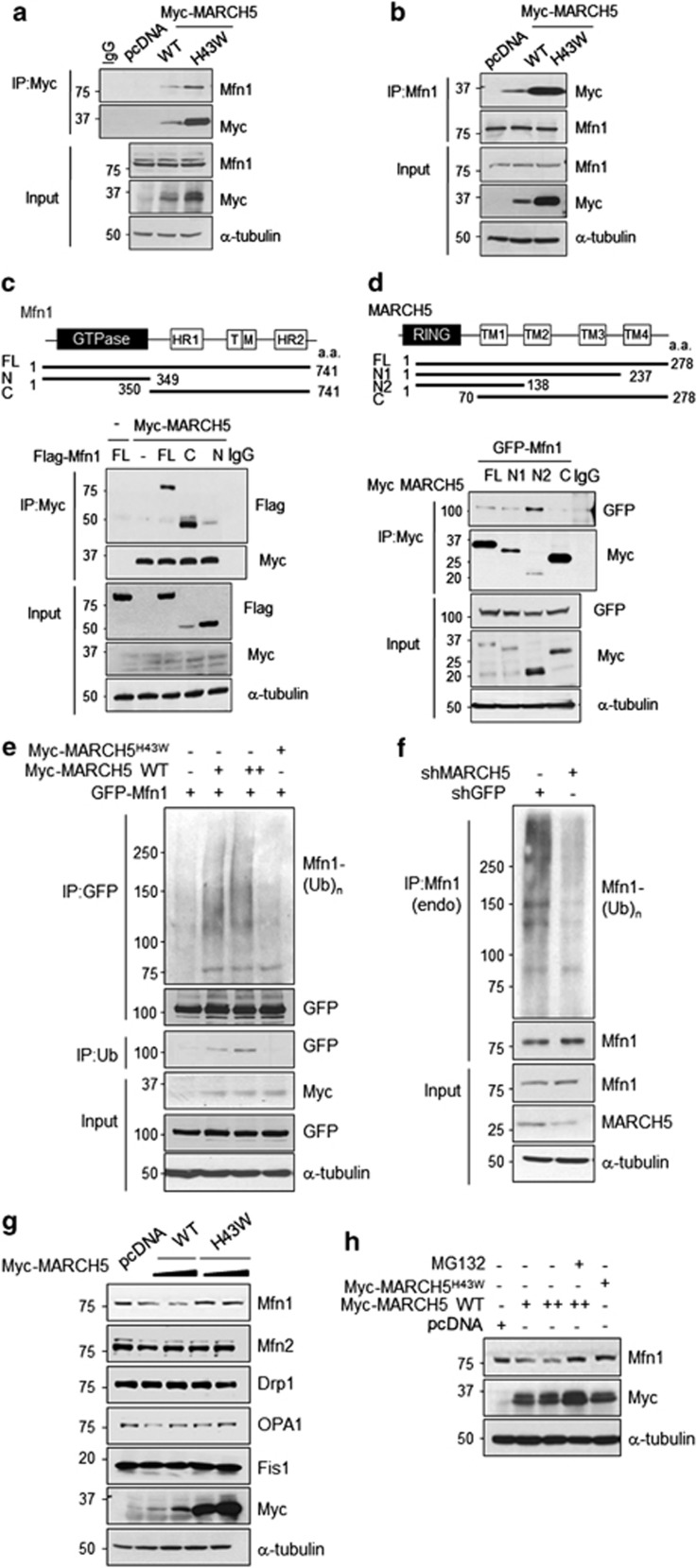
Mfn1 levels are controlled by MARCH5-mediated ubiquitylation. (**a**) HeLa cells were transfected with pcDNA, Myc-MARCH5 WT or Myc-MARCH5^H43W^ (a RING-domain mutant form lacking ligase activity) and same amount of proteins were immunoprecipitated with anti-Myc antibody. The interaction between MARCH5 and Mfn1 were determined by immunoblotting. (**b**) The lysates were reciprocally immunoprecipitated with anti-Mfn1 antibody and the interaction was determined by anti-Myc antibody. (**c**) 293T cells were co-transfected with plasmid of Myc-MARCH5 and Flag-Mfn1 full length (FL), Flag-Mfn1 N-terminus (N, 1–349) or C-terminus (C, 70–278). HR, heptad-repeat regions; TM, transmembrane. Lysates were immunoprecipitated with anti-Myc antibody. (**d**) GFP-Mfn1 and Myc-MARCH5 fragments (N1; 1–237, N2; 1–138 C; 70–278) were expressed in 293T cells and the lysates were immunoprecipitated with anti-Myc antibody. (**e**) HeLa cells were transfected with Myc-MARCH5 or Myc-MARCH5^H43W^ and GFP-Mfn1 and treated with 10 *μ*M of MG132 for 12 h before harvesting. For evaluating the levels of ubiquitylation of GFP-Mfn1, total cells were lysed and immunoprecipitated with anti-GFP antibody followed by analyzed immunoblotting with anti-ubiquitin antibody. (**f**) After GFP or MARCH5, shRNA-expressing HeLa cells were treated with 10 *μ*M of MG132 for 12 h. The lysates were immunoprecipitated with anti-Mfn1 (endogenous) antibody, the ubiquitylation levels were evaluated by immunoblotting with anti-ubiquitin antibody. (**g**) After cells were transfected with pcDNA, Myc-MARCH5 WT or Myc-MARCH5^H43W^, the protein levels were evaluated by using indicated antibodies. (**h**) Mfn1 expression levels were evaluated by immunoblotting in pcDNA, Myc-MARCH5 WT or Myc-MARCH5^H43W^-expressing cells, in absence or presence of proteasome inhibitor, MG132 (20 *μ*M, 4 h)

Hence, the question is how the amount of Mfn1 is controlled in different cellular environments. It has been shown that Mfn1 and Mfn2 are ubiquitinated by the cytosolic E3 ubiquitin ligase, Parkin, promoting selective removal of impaired mitochondria.^26, 31^ We found that Mfn1 levels can be changed in MARCH5-depleting cells.^19^ In this study, we evaluated whether MARCH5 is involved in the regulation of Mfn1 levels in the mitochondrial adaptation process. We first determined MARCH5 interaction with Mfn1 as well as ubiquitylation of Mfn1. Associations between MARCH5 and endogenous Mfn1 were detected by immunoprecipitation (IP) assay (Figure 4a). Reciprocal IP also showed the same binding patterns (Figure 4b). When the interaction domains of these proteins were examined, the C-terminal Mfn1 region comprising HR and TM domains was found to strongly interact with MARCH5 (Figure 4c). MARCH5 is composed of an N-terminal RING and 4 TM (Figure 4d) domains. The N2 mutant of MARCH5 interacts better with Mfn1 than the full-length MARCH5 (Figure 4d), an indication that a smaller N-terminal MARCH5 fragment anchored at the mitochondrial outer membrane is more accessible to Mfn1.

Next, we determined the effect of MARCH5 on Mfn1 ubiquitylation. When cells were transfected with GFP-Mfn1 and Myc-MARCH5, we observed that ubiquitin-conjugated GFP-Mfn1 increased in Myc-MARCH5-expressing cells, but not in Myc-MARCH5^H43W^-expressing cells (Figure 4e). IP using anti-ubiquitin antibody verified the ubiquitylation of GFP-Mfn1 in Myc-MARCH5-expressing cells, but not in Myc-MARCH5^H43W^-expressing cells (Figure 4e, middle panel). Inversely, the basal ubiquitylation of Mfn1 in proliferating cells was significantly reduced in MARCH5-depleted cells (Figure 4f). Consistent with these findings, we observed that Mfn1 levels were reduced by overexpression of wild-type (WT) MARCH5 but not by MARCH5^H43W^ (Figure 4g) and the degradation by MARCH5 was inhibited by MG132 (Figure 4h). In contrast, MARCH5 did not significantly affect other mitochondrial dynamics proteins including Mfn2 (Figure 4g), confirming that Mfn1 is a major target of MARCH5. These data clearly demonstrate that Mfn1 interacts with MARCH5 in cells and its expression is controlled by MARCH5 through ubiquitylation and subsequent degradation.

### MARCH5-dependent Mfn1 ubiquitylation and degradation are significantly enhanced under mitochondrial stress conditions

**Figure 5:**
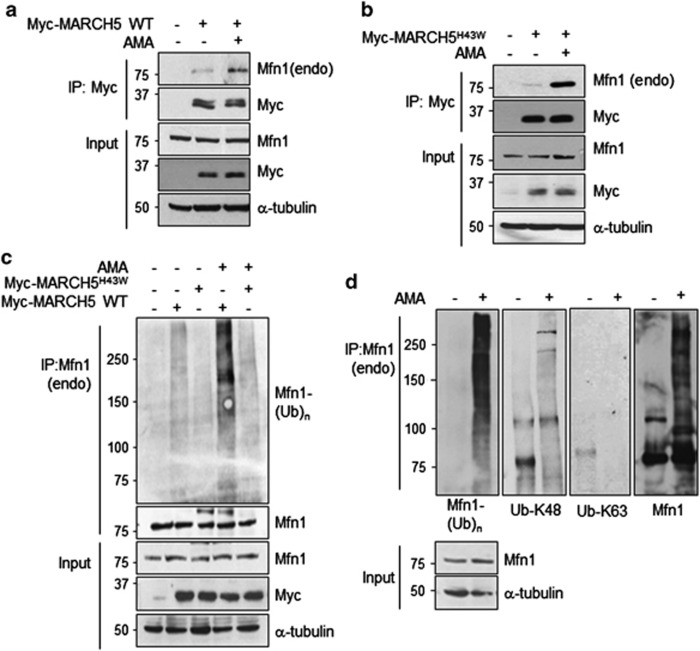
Under mitochondrial stress condition, the ubiquitylation of Mfn1 by MARCH5 is enhanced. (**a**) After HeLa cells were transfected with control or Myc-MARCH5 WT and 10 *μ*M of AMA was added to the cells for 7 h. Lysates were immunoprecipitated with anti-Myc antibody and the association between MARCH5 and Mfn1 was determined by immunoblotting. (**b**) Before harvesting, AMA was added to pcDNA or Myc-MARCH5^H43W^-expressing HeLa cells and the association between MARCH5^H43W^ and Mfn1 was evaluated by immunoblotting analysis. (**c**) Cells were transfected with pcDNA, Myc-MARCH5 WT or Myc-MARCH5^H43W^ and incubated with 10 *μ*M of AMA for 5 h and 20 *μ*M MG132 for 4 h. Lysates were immunoprecipitated with anti-Mfn1 (endogenous) antibody and the ubiquitylation levels of Mfn1 were evaluated by anti-ubiquitin antibody. (**d**) HeLa cells were treated with 10 *μ*M of AMA for 5 h and harvested. The lysates were immunoprecipitated with anti-Mfn1 (endogenous) antibody and analyzed by immunoblotting. The ubiquitylation levels of endogenous Mfn1 were evaluated using anti-ubiquitin antibody and anti-ubiquitin K48 or K63-specific antibody

Next, we examined whether MARCH5 affects the mitochondrial adaptation process under mitochondrial stress. As we observed only a little mitochondrial hyperfusion 3 h after AMA treatment and a considerable mitochondrial hyperfusion was shown at 7 h before cells fell into death (Figures 1b and c), we determined the interactions between Mfn1 and MARCH5 undergo changes upon AMA treatment. Remarkably, co-IP assay revealed a significant increase of MARCH5 binding to Mfn1 at 7 h (Figure 5a). In addition, binding of MARCH5^H43W^ to Mfn1 was also increased in the presence of AMA (Figure 5b), indicating that MARCH5 strongly controls Mfn1 levels upon AMA treatment. These findings were supported by a stronger ubiquitylation of Mfn1 by MARCH5 upon AMA treatment (Figures 5c and d). Notably, ubiquitylation of Mfn1 was dramatically increased in the presence of AMA (lanes 2 and 4, Figure 5c), whereas little ubiquitylation was found with MARCH5^H43W^ (Figure 5c; Supplementary Figure 4), demonstrating that acceleration of Mfn1 ubiquitylation under AMA stress is dependent on MARCH5 ligase activity. Using specific antibodies, we confirmed that augmented ubiquitylation of Mfn1 by AMA treatment was K48 linked, and not K63 linked (Figure 5d). The data strongly indicate that MARCH5-dependent Mfn1 ubiquitylation and degradation are significantly enhanced under mitochondrial stress, which suppresses an excessive accumulation of Mfn1 level in these cells.

### The acetylation-deficient K491R mutant of Mfn1 reduced its interaction with MARCH5 and subsequently diminished its MARCH5-dependent ubiquitylation

**Figure 6:**
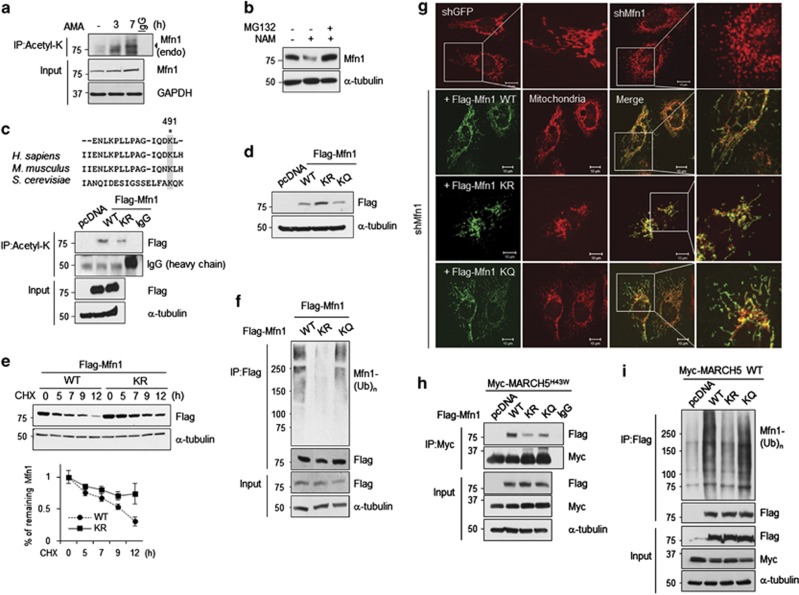
The K491R mutant of Mfn1 reduced its MARCH5-dependent ubiquitylation. (**a**) AMA exposed HeLa cells for indicated time were treated with MG132 before harvesting, the same amount of lysates were immunoprecipitated with anti-acetyl-Lys (K) antibody. The acetylation levels of Mfn1 were evaluated using anti-Mfn1 (endogenous) antibody. Arrowhead indicates acetylated Mfn1 (endogenous). (**b**) Endogenous Mfn1 levels of 293T cells were determined under treatment of deacetylase (sirt family) inhibitor, 50 mM of nicotinamide for 12 h, in absence or presence of MG132 (10 *μ*M). (**c**) Alignment of the Mfn1 protein sequences surrounding lysine 491 from yeast to human; conserved lysine residues are shaded (upper panel). Flag-Mfn1 WT or KR mutant expressing 293T cells were treated with MG132 before harvesting. Using the lysates, the acetylation status of Flag-Mfn1 WT or KR was evaluated by IP with anti-acetyl K antibody (lower panel). (**d**) The expression levels of Flag-Mfn1 WT, lysine 491 residue mutants, KR and KQ were evaluated by immunoblotting with anti-Flag antibody. (**e**) Cycloheximide (CHX) chase assays on Mfn1 stability. shMfn1 stably expressing HeLa cells were transfected with Flag-Mfn1 WT or KR, followed by treatment of CHX (100 *μ*g/ml) for indicated time. Relative levels of Mfn1 WT and KR were quantified and normalized to α-tubulin. Data represent average of three independent experiments. Error bars, mean±S.E.M. (**f**) Flag-tagged Mfn1 WT, KR or KQ mutant expressing 293T cells were immunoprecipitated with anti-flag antibody, followed by immunoblotting with anti-ubiquitin antibody. (**g**) Flag-Mfn1 WT, KR or KQ mutants were ectopically expressed in GFP or Mfn1 shRNA stably expressing HeLa cells. Mitochondria were visualized with MitoTracker Red (red) and Flag-Mfn1 was immunostained with Alexa 488 (green)-conjugated secondary antibody. Representative mitochondrial images were obtained by confocal microscopy and scale bars represent 10 *μ*m. (**h**) Myc-MARCH5^H43W^ was co-transfected with pcDNA, Flag-Mfn1 WT or lysine mutants (KR, KQ) into 293T cells and lysates were immunoprecipitated with anti-Myc antibody. The binding affinity between MARCH5 and Mfn1 lysine mutants were determined by immunoblotting. (**i**) Flag-Mfn1 WT, lysine 491 residue mutants (KR, KQ) were expressed with Myc-MARCH5 WT in 293T cells and treated with MG132 before harvesting. The ubiquitylation levels of Flag-Mfn1 WT, KR or KQ by MARCH5 WT were assessed by immunoblotting with anti-ubiquitin antibody after IP by anti-Flag antibody

E3 ubiquitin ligases often recognize post-translationally modified substrates through phosphorylation, acetylation and so on.^32, 33^ Post-translational modification of MARCH5 including acetylation was detected by LC-mass in our preliminary experiments. MARCH5 binds Mfn1 and these interactions appear to be enhanced under mitochondrial stress conditions (Figure 5). These results suggest that post-translational modification on Mfn1 may occur in these cells. To test this possibility, we determined the acetylation status of Mfn1 using an anti-acetylated Lys antibody and found that acetylated Mfn1 levels had accumulated in addition to AMA (Figure 6a; Supplementary Figure 5). Treatment with nicotinamide decreased the Mfn1 level, which was restored in the presence of MG132 (Figure 6b), indicating that the protein stability of Mfn1 is reduced by its acetylation. Several Lys residues of Mfn1 are conserved from yeast to humans. Among them, acetylated Mfn1 at K491 was identified as one of the binding partners of MARCH5 in tandem affinity purification-coupled mass analysis in which MARCH5 was used as a bait (data not shown). As shown in Figure 6c, IP using anti-acetylated-K antibody revealed that the acetylation-deficient K491R mutant of Mfn1 resulted in reduced acetylation. Expression levels of the K491R mutant were higher than those of WT and its acetylation mimetic K491Q mutant (Figure 6d). Next, we determined degradation rates of WT Mfn1 and K491R mutant were compared in the presence of CHX. The degradation of K491R mutant was delayed (Figure 6e). Consequently, the K491R mutant showed a significant reduction in Mfn1 ubiquitylation compared with the WT and K491Q mutants (Figure 6f). These results strongly suggest that acetylation at K491 of Mfn1 is important for its protein stability. Next, we tested whether the K491R mutant would alter the mitochondrial morphology by mimicking high Mfn1 level. As expected, cells deficient in Mfn1 displayed fragmented mitochondria and introduction of the K491R mutant into Mfn1 shRNA cells mainly resulted in aggregated mitochondria, whereas both WT and K491Q mutant of Mfn1 induced more elongated forms of the mitochondria (Figure 6g). We next tested whether the K491 site of Mfn1 is important for its binding to MARCH5. Compared with the WT Mfn1, the binding of MARCH5^H43W^ to the K491R mutant was reduced by three times (Figure 6h) and MARCH5-dependent ubiquitylation was also greatly reduced with the K491R mutant (Figure 6i). Thus, our results here for the first time demonstrate that acetylation of Mfn1 at K491 is important for stability and this is modulated by MARCH5-dependent binding and ubiquitylation.

### MARCH5^−/−^ MEF and MARCH5-knockout HeLa cells sensitized to cell death upon AMA treatment

**Figure 7:**
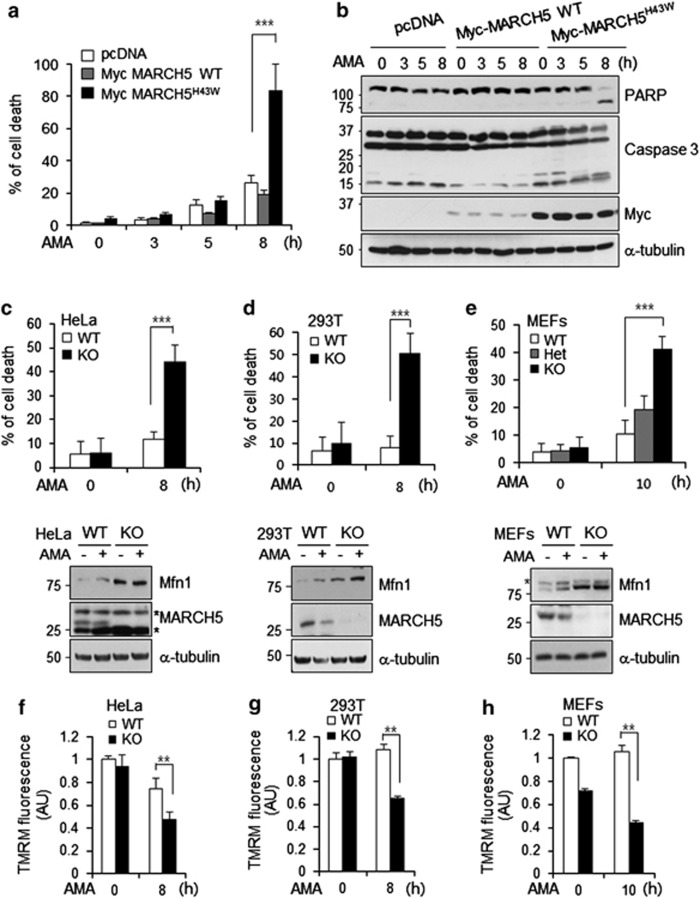
MARCH5^−/−^ MEFs and MARCH5-knockout HeLa cells sensitized to cell death upon AMA treatment. (**a**) After pcDNA, Myc-MARCH5 WT or Myc-MARCH5^H43W^ were transfected to HeLa cells, cells were exposed to 10 *μ*M of AMA for indicated time and dead cells were counted by trypan blue staining. Data represent average of three independent experiments. Error bars, mean±S.E.M. ***P<0.001 *versus* pcDNA by Student's *t*-test. (**b**) PARP cleavage and active caspase-3 were analyzed by immunoblotting. (**c**–**e**) After 10 *μ*M of AMA treatment to HeLa, 293T or MEF WT, Het or knockout (KO) (MARCH5^+/+^, MARCH5^+/−^ or MARCH5^−/−^) cells for indicated time, dead cells were counted by trypan blue staining (upper graphs). Data represent average of three independent experiments. Error bars, mean±S.E.M. ***P<0.001 *versus* MARCH5^+/+^ by Student's *t*-test. The cell lysates were analyzed for expression levels of Mfn1 or MARCH5 by immunoblotting. Asterisk indicates nonspecific band (bottom panel). (**f**–**h**) Mitochondrial membrane potential (ΔΨm) was assessed by FACS analysis after tetramethyl rhodamine methyl ester staining. MARCH5 WT or KO cells (HeLa, 293T or MEFs) were exposed to AMA, and depolarized mitochondria were analyzed. Graph represents quantification of relative tetramethyl rhodamine methyl ester fluorescence intensity (AU, arbitrary unit). Data represent average of three independent experiments. Error bars, mean±S.E.M. **P<0.01 *versus* MARCH5 WT by Student's *t*-test

Given that MARCH5-dependent quality control contributes to the adaptation process in cells by promoting cell survival, the MARCH5^H43W^ ligase mutant would weaken cell survival under AMA treatment. We therefore introduced either the MARCH5 WT or MARCH5^H43W^ into cells and compared the cell survival after AMA treatment. Eight hours after AMA treatment, cell death in control cells was ∼20%. We found that overexpression of MARCH5 did not significantly affect cell survival response upon AMA treatment. However, cell death in cells expressing MARCH5^H43W^ dramatically increased (up to ∼70%) within 8 h of AMA treatment (Figure 7a). These findings are consistent with the observed appearance of cleaved PARP as well as with the activated caspase-3 in MARCH5^H43W^-overexpressing cells exposed to AMA (Figure 7b). In order to verify the function of MARCH5 in the prosurvival response, we generated MARCH5-knockout cells. MARCH5^−/−^ HeLa and 293T cells were generated using transcription activator-like effector nuclease. In addition, MARCH5^+/+^, MARCH5^+/−^ and MARCH5^−/−^ MEFs were generated from E13.5 embryo. We found that cell death was significantly increased in both MARCH5^−/−^ cells in response to AMA treatment (Figures 7c and d). MEFs appeared to be more resistant to AMA. Although 10 *μ*M of AMA did not induce cell death in WT MEFs at 10 h, it drastically increased cell death in MARCH5^−/−^ MEFs. MARCH5^+/−^ MEFs showed a slight increase in cell death, which was not significantly different from the MARCH5^+/+^ MEF cells (Figure 7e). Immunoblotting showed Mfn1 expression levels as well as a lack of MARCH5 and in these cells (Figures 7c–e). We also observed that perinuclear mitochondrial aggregation was apparent in MARCH5^−/−^ HeLa cells, which experienced severe cell death upon AMA treatment. In addition, knockdown of Mfn1 in these cells partially reduced cell death (Supplementary Figure 6). We compared the mitochondrial membrane potentials in MARCH5^+/+^ and MARCH5^−/−^ cells in AMA-stressed cells. The fluorescence intensities of tetramethyl rhodamine methyl ester were maintained in MARCH5^+/+^ cells 8–10 h after AMA treatment, whereas those were significantly lower in MARCH5^−/−^ cells (Figures 7f–h), demonstrating that mitochondrial hyperfusion mediated through Mfn1 contributes to mitochondrial homeostasis. We concluded that MARCH5 severely affected cell survival under mitochondrial stress and, therefore, that functional MARCH5 serves as an important quality controller under mitochondrial stress conditions.

## Discussion

In this study, we demonstrated a novel mitochondrial adaptation process mediated by two mitochondrial molecules, Mfn1 and MARCH5. Cooperation of these molecules provides elasticity of mitochondria dynamics, which contributes to mitochondrial homeostasis and cell survival.

Mfn1 and MARCH5 work in three steps. First, Mfn1 is rapidly accumulated against mitochondrial stress, which induces mitochondrial hyperfusion. Although it has been shown that mitochondrial dynamics proteins are required for mitochondrial hyperfusion,^12, 13, 34, 35^ this is the first report showing that mitochondrial dynamics protein levels are also actively controlled (Figure 1). Second, during Mfn1 accumulation, MARCH5 binding to Mfn1 and its subsequent ubiquitylation on Mfn1 is significantly enhanced (Figure 5). Third, the process of MARCH5 binding to Mfn1 and its ubiquitylation changed depending on the acetylation status of Mfn1. Thus, acceleration of Mfn1 degradation by MARCH5 under stress is an important quality control system that inhibits mitochondrial aggregation and cell death (Figures 3 and 7). Thus, these processes are critical components for cell survival. The MARCH5-dependent acetylated Mfn1 degradation is the first evidence of cellular strategy for mitochondrial homeostasis as far as we know.

Mitochondrial fusion activity is generally beneficial to cells and it contributes to dilution of mutant mitochondrial DNA and excess ROS.^8^ In fact, mitochondrial fusion is required for the maintenance of the mitochondrial genome.^9^ In addition, transient mitochondrial hyperfusion is observed in cells exposed to different toxic agents,^12, 34^ cold stress,^35^ starvation^13^ and hypoxia,^36^ and considered as a mitochondrial adaptation process that is often linked to cell survival. Depending on different kinds of stimuli, different fusion or fission molecules can be involved. In cells exposed to toxic agents such as CHX and actinomycin D, mitochondrial hyperfusion is mediated through long-form optic atrophy 1, SLP-2 and Mfn1.^12^ Enhanced mitochondrial fusion activity was shown to be obtained by Mfn2 that protects against cold stress-induced cell injury.^35^ In hypoxia conditions, some cancer cells exhibited an enlarged mitochondrial phenotype partly mediated through Mfn1.^36^ All these results clearly indicate that mitochondrial dynamics proteins are deeply involved in cellular adaptation process to different stresses. However, whether expression levels of these mitochondrial dynamics proteins are changed or modified in response to stress have not been fully addressed. So far, phosphorylation of Drp1 during starvation has been well recognized.^13^ In the present study, we showed that Mfn1 expression levels are tightly controlled under mitochondrial stress. Thus, it can be speculated that cells develop different strategies utilizing mitochondrial dynamics proteins for effective adaptation or cell survival in response to stresses.

We demonstrated that Mfn1 is a major target of MARCH5 (Figures 4 and 5). As the Mfn1 level accumulated in response to AMA treatment (Figures 1c and d), it had been reasonable to assume that the MARCH5 interaction with Mfn1 would have been weakened at the condition Mfn1 accumulation. However, paradoxically, MARCH5-dependent Mfn1 degradation was significantly enhanced under mitochondrial stress conditions (Figure 5). We therefore speculate that under mild mitochondrial stress condition, initial Mfn1 accumulation and mitochondrial hyperfusion help cells adapt to the stress and maintain viability. Excessive Mfn1 levels threaten cell viability (Figures 3c and d); therefore, if Mfn1 accumulation continues, MARCH5 reduces the Mfn1 levels through intensive ubiquitylation. Thus, MARCH5 under stress functions as a quality control system that inhibits mitochondrial aggregation and cell death. This characterization is also supported by the increased cell death observed in cells expressing MARCH5^H43W^ (Figures 7a and b). In yeast, increased levels of fizo1p induce mitochondrial aggregation and failure to respire.^37^ In mammalian cells, perinuclear mitochondrial aggregates were also found by overexpression of *Mfn* genes^38^ or when endothelial cells were exposed to hypoxia.^39^ It seemed that excessive mitochondrial hyperfusion leading to perinuclear mitochondrial cluster sensitized cells to apoptotic cell death or accumulated ROS levels in the nucleus.^39^ In addition, accumulation of Mfn1 in MARCH5-depleted cells promoted cellular senescence.^19^ Thus, cells may evolve MARCH5 to control Mfn1 levels in the range which do not disturb cell function.

Here we also demonstrated that binding of MARCH5 to Mfn1 and its subsequent ubiquitylation changes depending on the acetylation status of Mfn1 (Figure 6; Supplementary Figure 3). To our knowledge, this is the first report showing that acetylation of Mfn1 actually controls its stability through its binding to E3 ligase. Recently, it was reported that phosphorylation on Mfn2 by JNK triggered Mfn2 degradation through another ubiquitin ligase, Huwe1.^32^ Acetylation of endogenous Mfn1 was weak but increased under mitochondrial stress (Figure 6a). It was noted that Mfn2 can be phosphorylated by both PINK and JNK under different stress conditions.^27, 32^ The phosphorylation on Mfn2 by JNK recruits Huwe1, triggering Mfn2 degradation and mitochondrial fragmentation,^32^ whereas another phosphorylation on Mfn2 by the PINK1 site contributes to recruitment of Parkin at the mitochondria.^27^ Thus, it is likely that cells utilize different post-translational modifications on the mitochondrial fusion machinery to respond to different cellular stresses. In summary, we proposed a novel stress response pathway mediated by MARCH5 and Mfn1. MARCH5-mediated quality control on acetylated Mfn1 facilitates mitochondrial homeostasis and prosurvival responses under mitochondrial stress conditions.

## Materials and Methods

### Cell culture and transfections

HeLa, HeLa S3 and 293T were purchased from the ATCC. Mfn1^−/−^ and WT MEFs have been previously described.^4^ MARCH5-knockout HeLa and 293 cells were generated by using transcription activator-like effector nucleases technology. The *MARCH5*-specific transcription activator-like effector nuclease plasmids were obtained from ToolGen Inc. (Seoul, Korea).^40^ MARCH5^−/−^ MEFs were generated from C57BL/6 MARCH5^flox/flox^ embryo. Cells were cultured in Dulbecco's modified Eagle's medium (Invitrogen, Carlsbad, CA, USA) supplemented with 10% heat-inactivated fetal bovine serum, 1% penicillin and streptomycin (Gibco, BRL, Grand Island, NY, USA) in a 5% CO_2_ incubator at 37 °C. Plasmid DNA constructs were transfected by using polyethylenimine (Polysciences, Inc., Warrington, PA, USA) as previously described.^19^ For shRNA, 1 day after transfection, cells were grown in the media containing 200 *μ*g/ml hygromycin B (Roche Diagnostic, Indianapolis, IN, USA) for 36 h to select transfected cells. Dying cells were removed by brief centrifugation and the survived cells were re-seeded on culture plates, which is designated as day 0 and further grown in 30 *μ*g/ml hygromycin B containing Dulbecco's modified Eagle's medium.^19^

### Plasmid construction

Myc-tagged MARCH5-expressing vector was generated by using MARCH5-YFP and MARCH5^H43W^-YFP as described in Karbowski *et al.*^20^ To generate GFP-Mfn1 WT, PCR was carried out with GFP-Mfn1^T109A^ as a template using the following primers: 5′-ATGGCAGAACCTGTTTCTCCACTGAA-3′and 5′-GGAAGCAATTGGTTATATGG CCAATCCCA-3′. The PCR fragments were cloned into *Xho*I and *Mfe*I site of GFP-Mfn1^T109A^. GFP-Mfn1^T109A^ expression vector was provided by Magaret T. Fuller (Stanford University, CA, USA). Silencing of endogenous MARCH5 mRNA was carried out using shRNA system.^20^ Mfn1 shRNA were generated using method previously described.^19^

### Immunocytochemistry and microscopy analysis

To visualize mitochondria, 125 nM of MitoTracker Red (Molecular Probes, Eugene, OR, USA) was added to cultured media and incubated cells for 30 min at 37 °C. After cells were washed with phosphate-buffered saline for three times, the mitochondria-stained cells were fixed with 4% paraformaldehyde solution for 10 min and rinsed with a mixture of phosphate-buffered saline: methanol (1 : 1). The fixed cells were permeabilized with methanol for 20 min at −20 °C. For immune fluorescence staining, cells were blocked with 1% bovine serum albumin in phosphate-buffered saline for 1 h at room temperature, followed by incubation with appropriate primary antibodies (anti-c-Myc antibody) overnight at 4 °C, and subsequently immunostained with an Alexa 488-conjugated secondary antibody. For mitochondria imaging in live cells, cells were seeded in a coverglass-bottom dish (SPL Life Sciences, Pochun, Korea) and mitochondria were stained with 100 nM of MitoTracker Green (Molecular Probes) for 30 min. Images were captured and analyzed using LSM 710 Confocal Microscopy (Carl Zeiss, Welwyn Garden City, UK). For time course analysis, after HeLa cells were transfected with YFP-mito and treatment of different concentrations of AMA, images were analyzed after 3 h of AMA treatment using fluorescence time-lapse microscope (Nikon eclipse, Nikon, Tokyo, Japan).

### IP and immunoblotting

For IP assays, cells were sonicated in IP buffer (50 mM HEPES, pH 7.5, 150 mM sodium chloride, 0.1% NP-40, 5 mM EDTA, 1 mM DTT and protease inhibitor). Protein lysates (500∼1000 *μ*g) were immunoprecipitated with 1 *μ*g of indicated antibodies at 4 °C overnight with agitation. The protein–antibody complex was further incubated with 25 *μ*l of protein A-sepharose beads (GE Healthcare Bio-Sciences AB, Uppsala, Sweden) for 1 h 30 min. After beads were washed with IP buffer four times, beads were heated in 2 × sample buffer before separating by SDS-PAGE and were transferred to the polyvinylidene difluoride membrane (Millipore, Billerica, MA, USA) or nitrocellulose membrane (Millipore). The immunoblots were visualized by enhanced chemiluminescence system (Amersham Biosystems, Foster City, CA, USA).

### *In vivo* ubiquitinylation assay

Cells were treated with 10 *μ*M of MG132 for 4∼12 h before harvest. Whole-cell extracts were lysed by sonication in IP buffer (50 mM HEPES, pH 7.5, 150 mM sodium chloride, 0.1% NP-40, 5 mM EDTA, 1 mM DTT and protease inhibitor). Lysates were clarified by centrifugation and quantified by using a Bradford reagent (Bio-Rad Laboratories, Foster City, CA, USA). The same amount of protein lysates (500∼1000 *μ*g) were immunoprecipitated with anti-GFP or anti-Mfn1 antibody coupled to protein G-sepharose beads for 1 h 30 min at 4 °C. The immune complex was washed four times with IP buffer thoroughly. The samples were dissolved in 2 × sample buffer, boiled for 5 min and separated by SDS-PAGE. The ubiquitylation level was analyzed by immunoblotting using anti-ubiquitin antibody (Santa Cruz Biotechnology, Santa Cruz, CA, USA).

### Reagents and antibodies

AMA, cyclohexamide (CHX) and MG132 were obtained from Sigma-Aldrich (St. Louis, MO, USA). The Mitofusin 1 (Mfn1) antibody was purchased from Proteintech (Wuhan, China). Anti-ubiquitin, anti-GFP and anti-c-Myc were from Santa Cruz Biotechnology, anti-PARP antibody was purchased from Invitrogen. Anti-ub-K48 and anti-ub-K68-specific antibodies were purchased from Millipore.

### RT-PCR and real-time PCR

Total RNA was isolated from HeLa cells using TRIZOL reagent (Invitrogen). For reverse transcription reaction, 1 *μ*g of RNA was used to synthesize cDNA with Prime-Script RT reagent kit (Takara, Shiga, Japan). Two present of RT product was used for PCR analysis. The following primer sets were used. *MFN1*, forward 5′-ATGGCAGAACCTGTTTCTCC ACTGAA-3′, reverse 5′-GGAAGCAATTGGTTATATGGCCAATCCCA-3′ *GAPDH*, forward 5′-GCCTCAAGATCATCAGCAATGCCT-3′, reverse 5′-AGACCACCTGGTGCTCAGTGTAG-3′. Real-time PCR was carried out using CFX96 TouchTM Real-Time PCR Detection System (Bio-Rad Laboratories). The primer set used was *Mfn1*, forward 5′-AGACTGAGCTGGACCACCCATG-3′, reverse 5′-TTAGGATTCTTC ATTGCTTGAAGGTAGA-3′.

### Trypan blue counting

After cells were treated with AMA for indicated time, cells were collected with brief centrifugation. Collected cells were suspended with phosphate-buffered saline and mixed at 1 : 1 dilution of the suspension using a 0.4% trypan blue solution (Sigma-Aldrich). After incubating the mixture for 1 min, both the number of stained cells and total cells were counted by using hemocytometer. The calculated percentages of stained cells were represented as the percentage of death cells.

### Statistical analysis

In each result, the error bars represent the mean±S.E.M. from at least three independent experiments. The statistical significance was performed with two-sided unpaired Student's *t*-test. *P*-values are indicated in the legends.

## Abbreviations

AMA: antimycin A
CHX: cycloheximide
MARCH5: membrane-associated RING-CH
MEF: mouse embryonic fibroblast
