# Supplementary figures and images for: MARCH5-mediated quality control on acetylated Mfn1 facilitates mitochondrial homeostasis and cell survival
Source: Cell Death Dis. 2014 Apr 10;5(4):e1172–. doi: 10.1038/cddis.2014.142 (PMC5424118; doi:10.1038/cddis.2014.142)

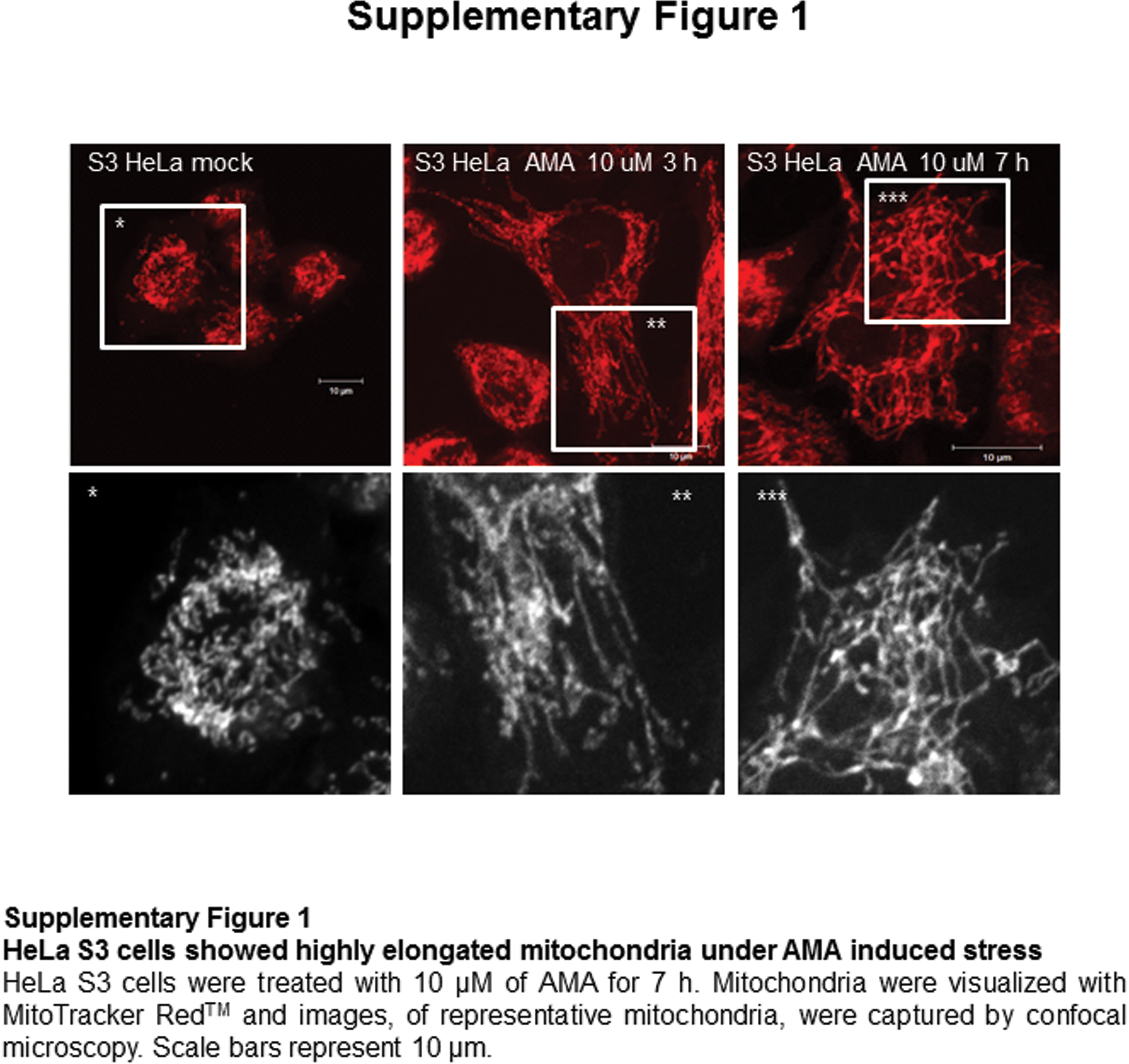

Supplement: Supplementary Figure 1 [file cddis2014142x1.tif]

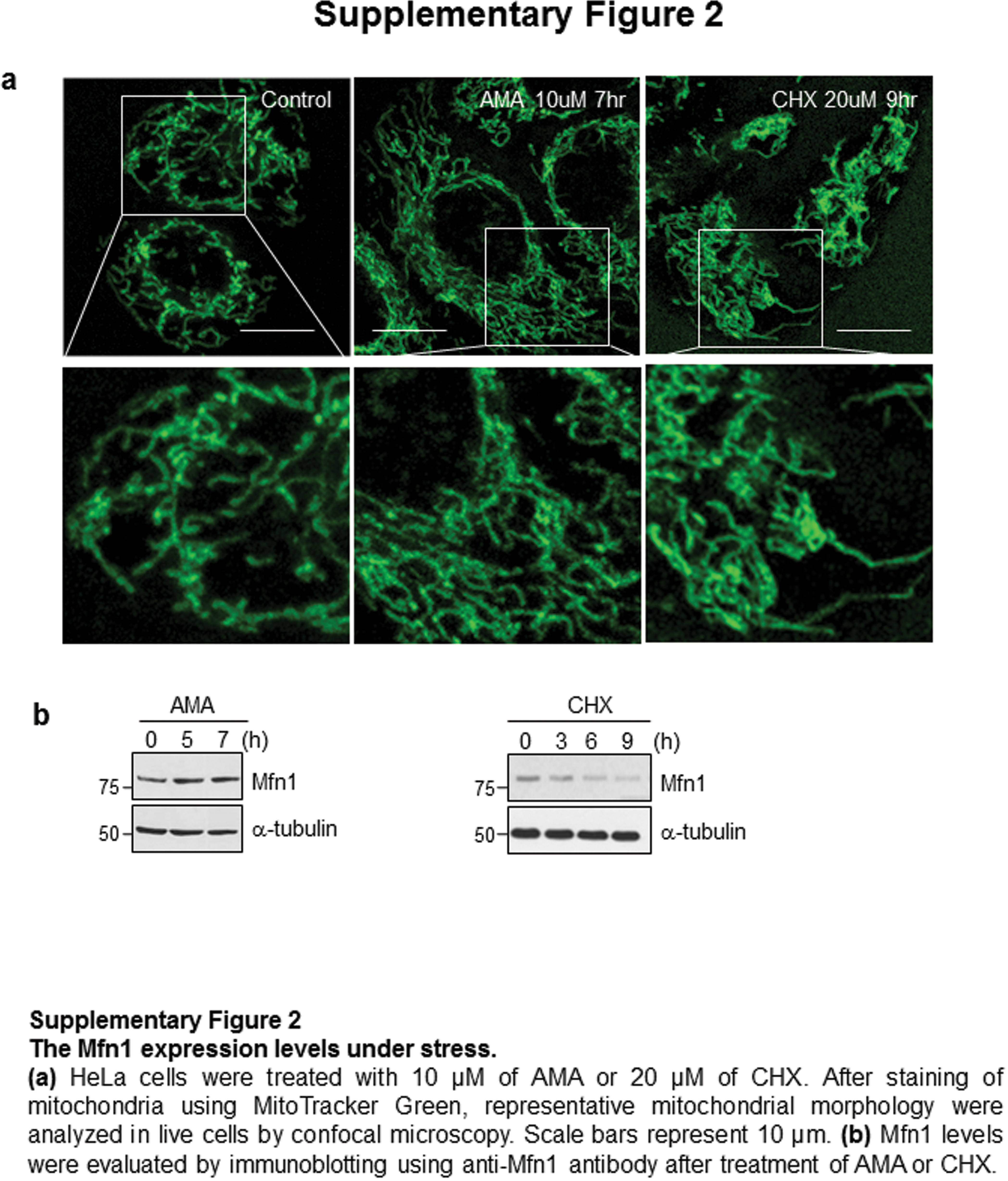

Supplement: Supplementary Figure 2 [file cddis2014142x2.tif]

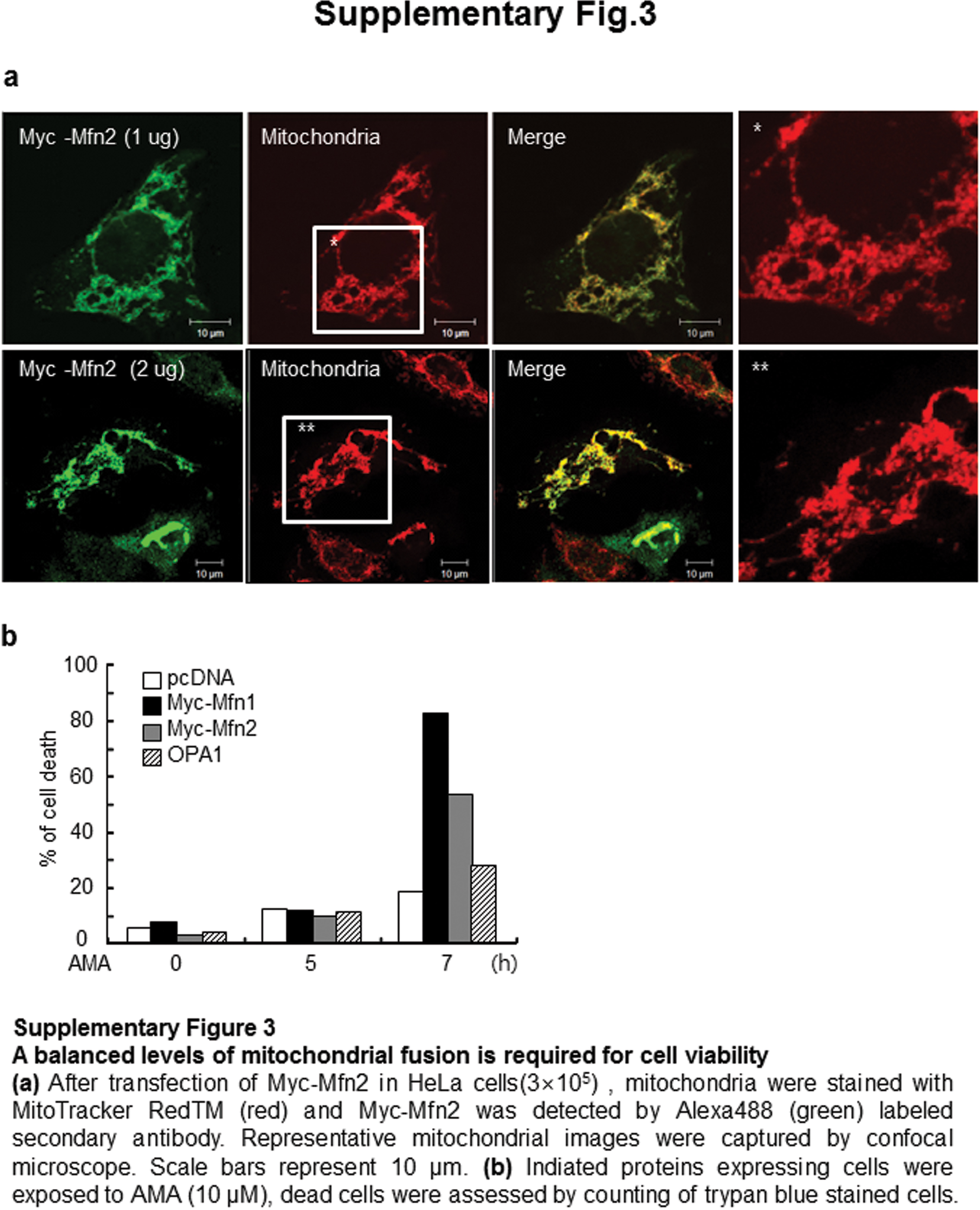

Supplement: Supplementary Figure 3 [file cddis2014142x3.tif]

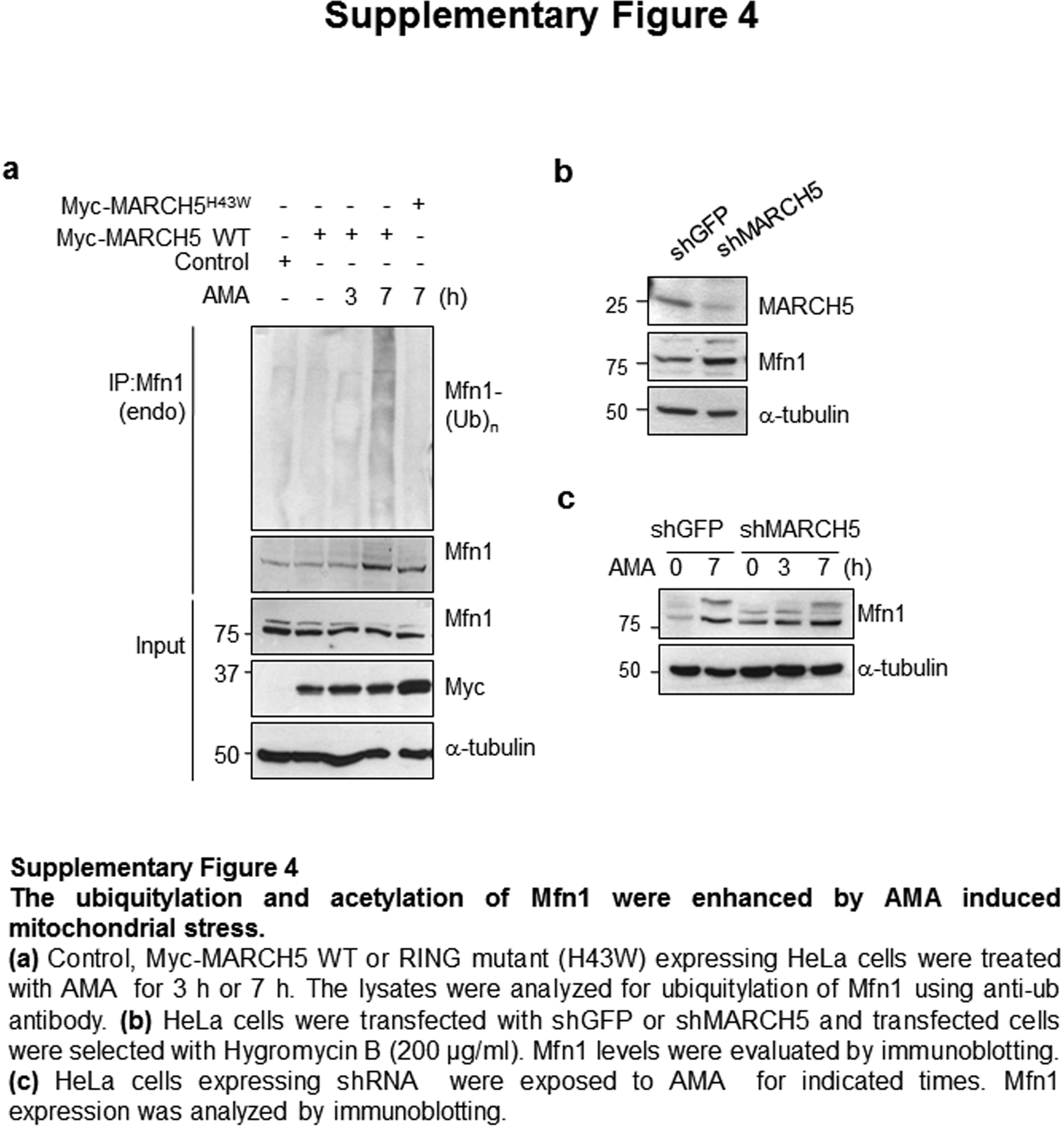

Supplement: Supplementary Figure 4 [file cddis2014142x4.tif]

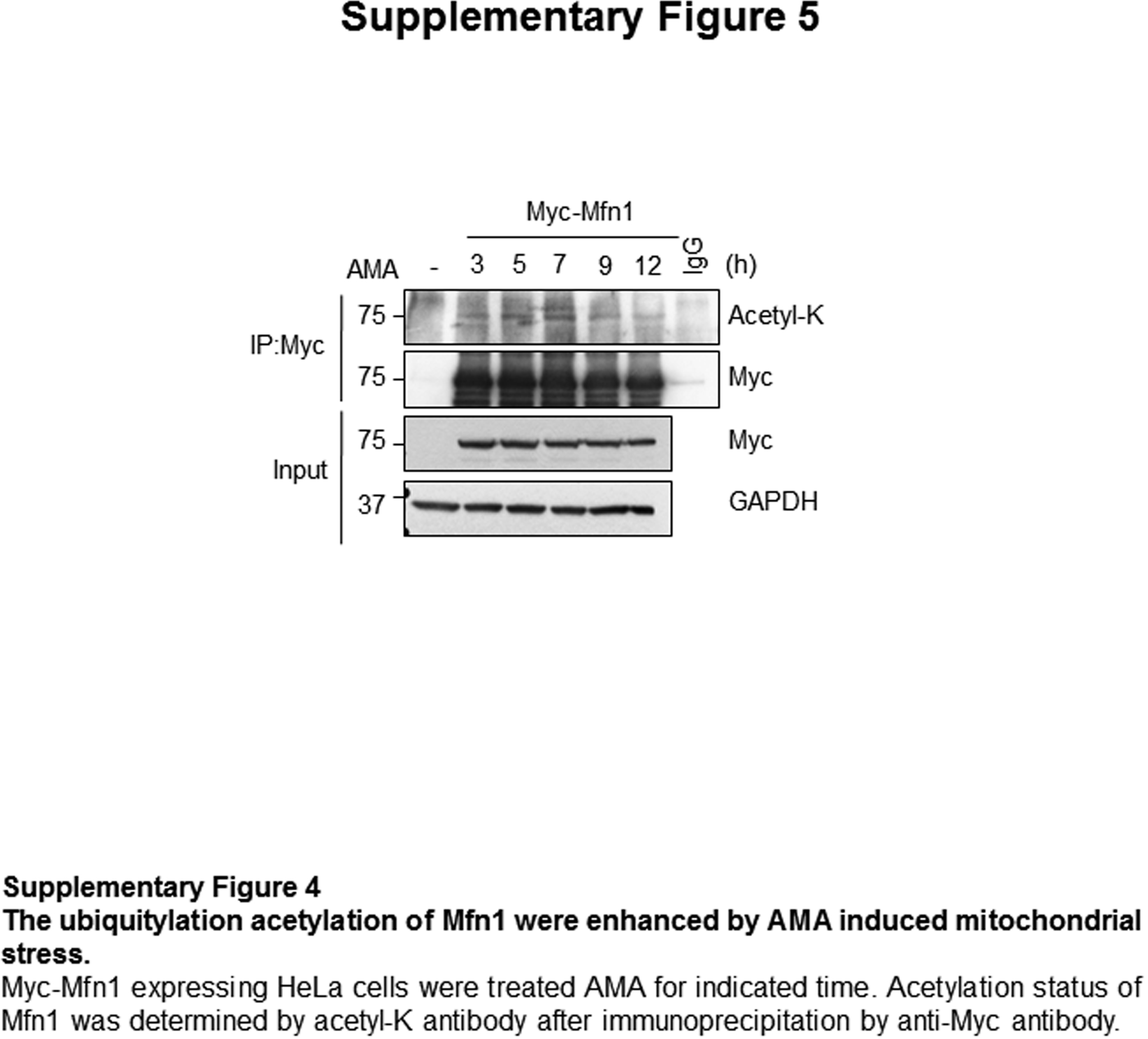

Supplement: Supplementary Figure 5 [file cddis2014142x5.tif]

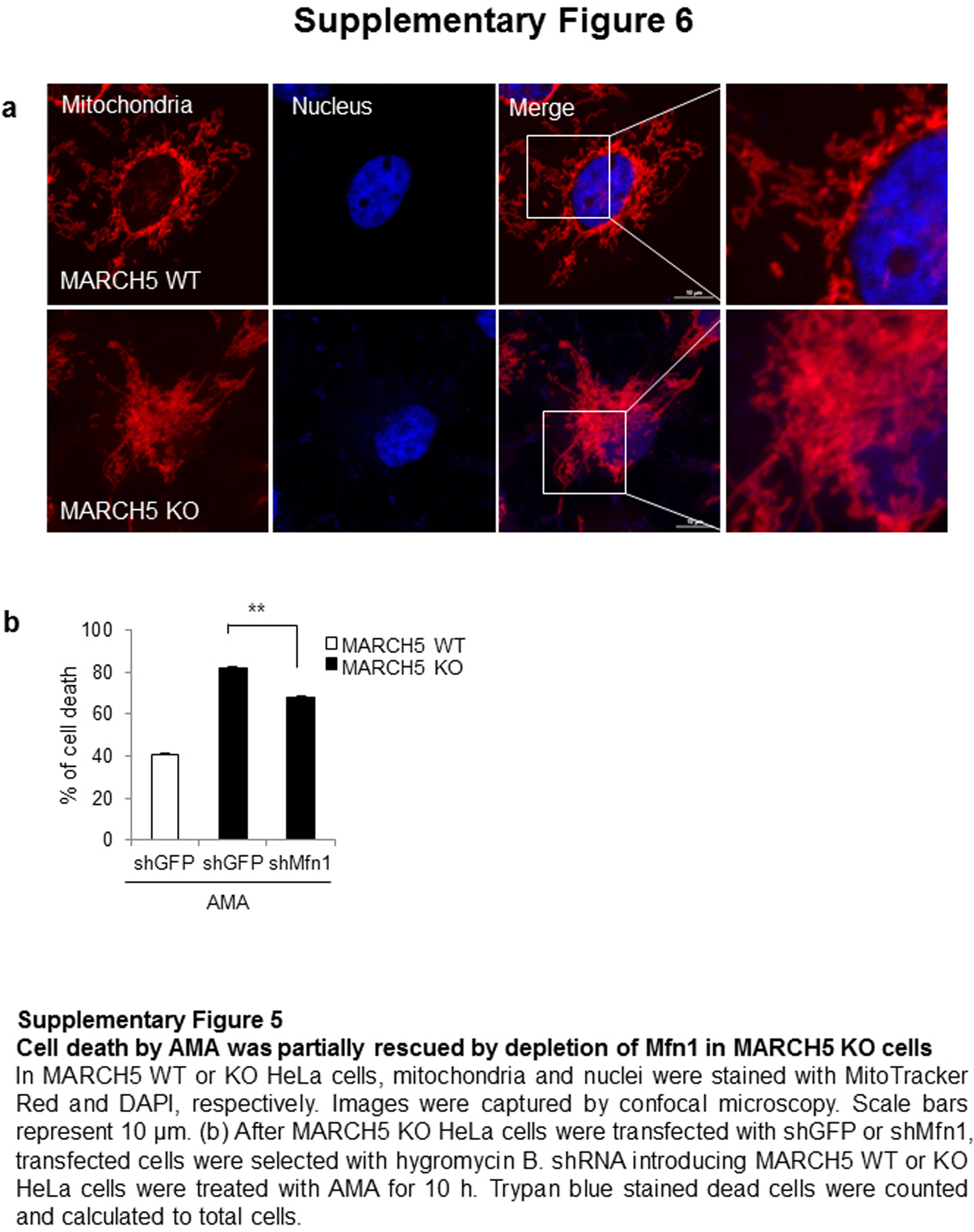

Supplement: Supplementary Figure 6 [file cddis2014142x6.tif]

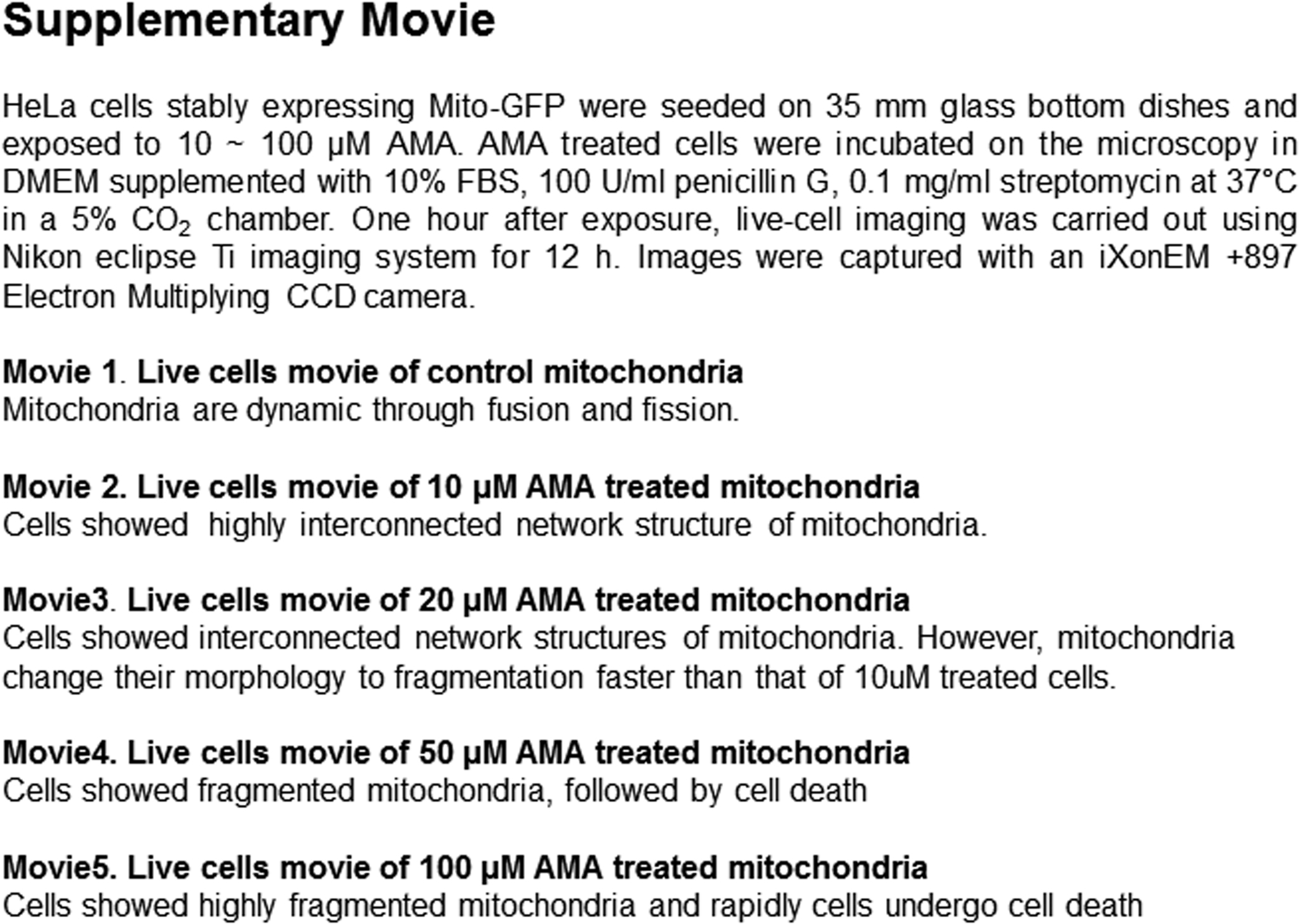

Supplement: Supplementary Movie Information [file cddis2014142x12.tif]
